# Influence of Honey Varieties, Fermentation Techniques, and Production Process on Sensory Properties and Odor-Active Compounds in Meads

**DOI:** 10.3390/molecules29245913

**Published:** 2024-12-14

**Authors:** Daria Cicha-Wojciechowicz, Natalia Drabińska, Małgorzata Anna Majcher

**Affiliations:** Faculty of Food Science and Nutrition, Poznań University of Life Sciences, Wojska Polskiego 31, 60-624 Poznań, Poland; daria.cicha@up.poznan.pl (D.C.-W.); natalia.drabinska@up.poznan.pl (N.D.)

**Keywords:** mead aroma, sensory analysis, odor-active compounds, gas chromatography, olfactometry

## Abstract

This study investigates the impact of key factors on the formation of odorants and sensory properties in mead. The effects of the honey type (acacia, buckwheat, linden), wort heating, and the fermentation method (commercial *Saccharomyces cerevisiae* yeasts, spontaneous fermentation, *Galactomyces geotrichum* molds) were examined. Twelve model mead batches were produced, matured for 12 months, and analyzed using gas chromatography–olfactometry (GC–O) and headspace SPME-GC/MS to identify odor-active compounds. Results confirmed that the honey type plays a significant role in sensory profiles, with distinct aroma clusters for buckwheat, acacia, and linden honey. Compounds like phenylacetic acid, 2- and 3-methylbutanal, and butanoic acid were identified as the most important odorants, correlating with sensory attributes such as honey-like, malty, and fermented aromas. Univariate and multivariate analyses, followed by correlation analysis, highlighted how production parameters affect mead aroma, providing insights to optimize sensory quality.

## 1. Introduction

Mead, an ancient alcoholic beverage made from honey, water, and yeast, has attracted considerable consumer interest for its unique and attractive aroma. It also serves as a captivating research subject for many scientists, who are particularly interested in understanding the factors that influence the development of mead’s characteristic aroma. Studies indicate that several factors can affect the aroma of mead, with one of the most frequently studied being the variety of honey used in its preparation. Li and Sun (2019) compared the volatile compound profiles of meads prepared from vitex, acacia, multi-floral, linden, and jujube honey. In their studies, they showed that the honey type had a significant impact on the abundance of alcohols, esters, and acids, which were the main groups of volatiles identified among meads [1]. Differences in meads prepared from chestnut, lime, and honeydew varieties of honey were also described by Vidrih and Hribar (2007) [2]. The type of honey can also influence other chemical parameters of mead, such as antioxidant capacity and phenolic compounds profile [3].

The fermentation of honey wort is one of the most important steps in mead production. Many important odor-active compounds such as esters, alcohols, ketones, or acids are produced by yeast activity. *Saccharomyces cerevisiae* are the most popular yeasts used in mead production, although a few studies were conducted to characterize aroma profiles of meads produced mostly with the use of non-*Saccharomyces* strains. In the study on mead prepared from multi-floral honey and rosehips, three yeast strains (two *S. cerevisiae* (var. *bayanus*) and *Torulaspora delbrueckii*) were employed. This study revealed that the yeast strain used for mead preparation can significantly affect volatile concentrations in the product, especially with the use of *T. delbrueckii* [4]. Similar experiments concerning the use of different yeast strains were also conducted by other researchers [5,6]. Recently, there has been a growing desire to improve the sensory properties of fermented beverages by using new species of microorganisms, such as molds. One of the examples is *Galactomyces geotrichum* mold, which, according to our previous studies, has the ability to improve the formation of odorants 2-phenylethan-1-ol, phenylacetic acid, and 2-phenylacetaldehyde with strong rose-like, honey aroma [7]. These molds are also capable of synthesizing volatile compounds such as 3-methylbutan-1-ol and 2-phenylethan-1-ol, which can significantly influence the aroma profile of alcoholic beverages [8].

Another factor that may affect the final attributes of mead is the heating of honey wort. This step may improve fermentation by inactivation of naturally occurring microbiota, which is represented by lactic acid bacteria groups and wild yeast strains [9]. On the other hand, wort boiling may increase the concentration of some off-flavors, such as 4-methylphenol, which smells like a horse stable [10]. Starowicz and Granvogl (2022) applied the molecular sensory science concept to verify the effect of wort boiling on the sensory analysis and volatile formation of meads. Although the authors compared the OAV of both meads, they did not discuss which odorants could be responsible for the difference in the sensory profiles [11]. Czabaj et al. (2017) showed that heat treatment of honey wort is highly correlated with the total antioxidant capacity and total phenolic content of meads, as the highest antioxidant capacity and phenolic content were observed for gently boiled mead samples [12].

In our previous studies, we successfully identified and quantified the key odorants of triple mead purchased from the producer at each production step, providing valuable insights into the evolution of these compounds during the production process [13]. These molecular-level findings have laid the groundwork for further investigation into the factors that influence the formation of key odorants in mead, which play a crucial role in shaping the sensory properties of the beverage. Consequently, the studies presented in this research aim to delve deeper into this subject by conducting model studies to elucidate the impact of various elements not only on the formation of important odorants in mead but also on its sensory properties. By systematically examining these variables, it is possible to enhance our understanding of the mechanisms driving the development of mead’s characteristic aroma and to identify potential strategies for optimizing its sensory qualities.

To the best of our knowledge, this study represents the first comprehensive identification and quantification of odorants in mead using gas chromatography–olfactometry (GC–O), while simultaneously examining numerous variables in the production process. This provides a detailed analysis of how these factors influence the overall aroma profile and which one has the greatest potential to change the mead’s aroma profile. For the first time, *G. geotrichum* molds have been utilized in the production of mead. This species was recently described as a fermenter of glucose and xylose and showed the ability of ethanol production from sugars present in the sugarcane bagasse hydrolysate [14,15]. Given their known ability to grow on glucose and galactose and their resistance to different ethanol and glucose concentrations (up to 14% ethanol and 65% glucose), there is a potential to harness these molds for improving the sensory properties of mead, introducing the novel method of the enhancement of rose-like, floral notes [16]. This innovative approach represents a significant advancement in the enrichment of mead aroma.

Numerous studies have investigated the impact of specific factors on either the sensory properties or the volatile composition of mead. However, to the best of our knowledge, no research has simultaneously investigated such a vast range of production parameters on both the sensory profile and aroma-active compounds of meads. Such comprehensive studies are not only scientifically significant but also hold practical value for mead producers. Understanding the changes in sensory properties during production is crucial for ensuring that the final product meets consumer expectations and preferences, thereby enabling the design of products with specific, desired features.

In detail, we investigated the influence of three critical factors: the type of honey (acacia, buckwheat, and linden honey), the effect of wort heating, and the type of fermentation (using commercial *S. cerevisiae* yeasts, spontaneous fermentation, and the use of *G. geotrichum* molds). Twelve model batches of mead were produced (including 12 months maturation) and their Solvent-Assisted Flavor Evaporation (SAFE) extracts were screened for odor-active compounds using gas chromatography–olfactometry. Following this, quantitation of the selected odorants using headspace SPME-GC/MS has been performed. Finally, to decode how each production parameter affects the final aroma of the mead, both univariate and multivariate analyses were applied, followed by the correlation analysis between the important odorants and the sensory attributes.

## 2. Results and Discussion

### 2.1. Quantitative Profile Sensory Analysis

**Figure 1:**
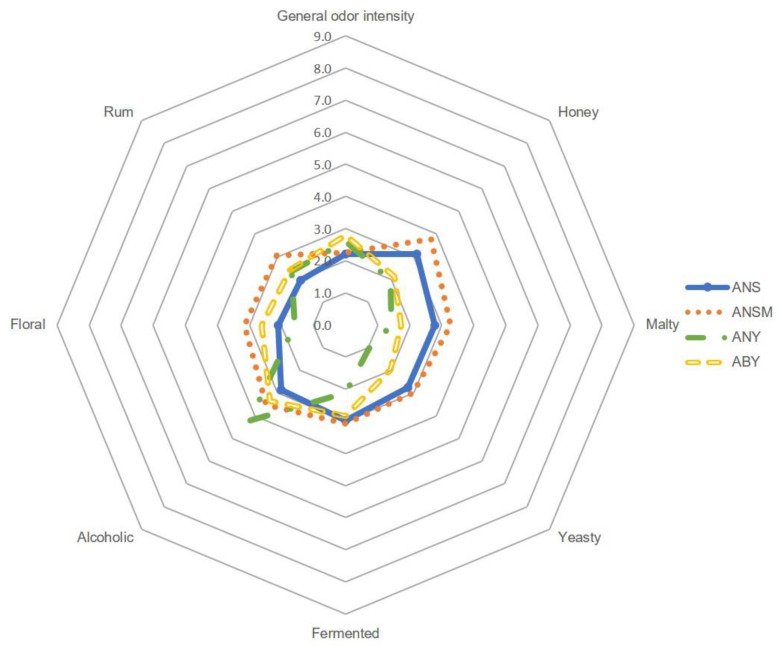
Quantitative olfactory profile analysis of acacia-based meads.

**Figure 2:**
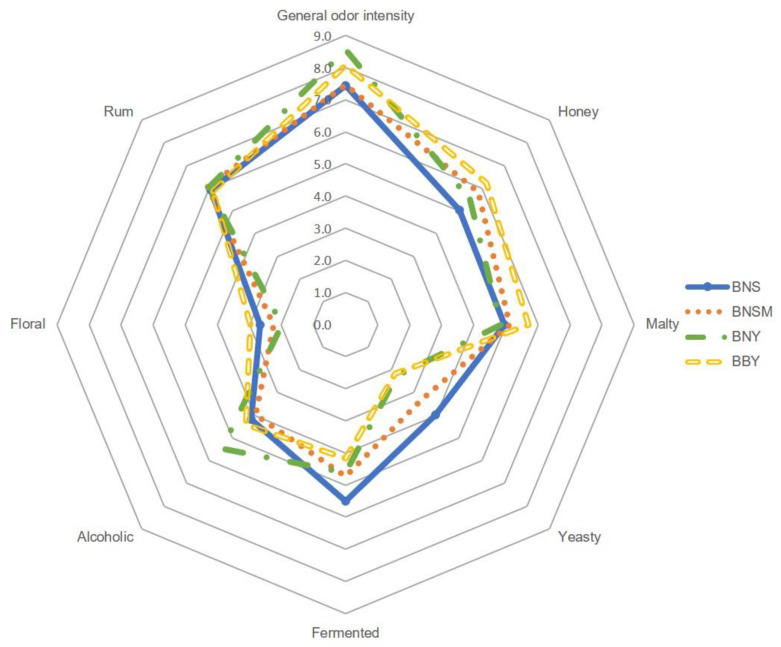
Quantitative olfactory profile analysis of buckwheat-based meads.

**Figure 3:**
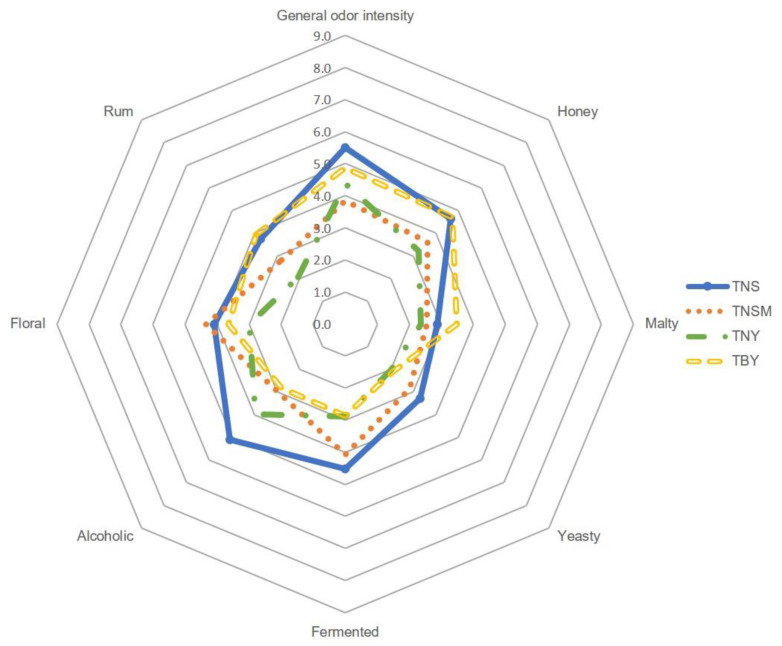
Quantitative olfactory profile analysis of tilia-based meads.

In order to provide a comprehensive understanding of the aromatic profile of all twelve model mead products, quantitative profile sensory analysis was carried out by a qualified team of experts. The results of the sensory evaluation, categorized by the type of honey used in the preparation, are shown in Figure 1, Figure 2 and Figure 3.

It has been noted that the general odor intensity of meads prepared from the same types of honey was very similar in each group. Evident differences can be noted between meads from different honey sources not only in overall aroma intensity but also in individual descriptors. All buckwheat-based meads (BNS, BNSM, BNY, and BBY) were noted for the highest general odor intensity (from 7.5 to 8.6), followed by tilia-based meads (TNS, TNSM, TNY, and TBY, score range from 3.8 to 5.5). The lowest values for general odor intensity were noted for acacia-based meads (ANS, ANSM, ANY, and ABY) with scores ranging from 2.2 to 2.8. Based on the profile sensory analysis, the buckwheat mead aroma was dominated by such attributes as honey (maximum score 8.6 for BNY), malty (maximum score 6.2 for BBY), rum (maximum score 6.1 for BNSM), fermented (maximum score 5.5 for BNS), and alcoholic (maximum score 5.5 for BNY). Floral and yeasty were slightly less perceptible (maximum scores of 3.0 and 4.0 for BBY and BNS, respectively). On the other hand, the sensation of floral aroma was more pronounced in tilia and acacia meads and exceeded that of rum, which was dominant in buckwheat mead. Considering the effect of wort boiling on the final aroma of the meads, it has been seen that boiling increased the honey and malty sensation, while it decreased the alcoholic aroma sensation. This effect has been observed in buckwheat and linden meads but not so much in acacia. Meads prepared from acacia honey showed greater variability in the aroma caused by the different types of fermentation. As shown in Figure 1, spontaneous fermentation, both with and without *G. geotrichum*, gave higher scores in honey, malty, and yeasty descriptors. In general, all meads with spontaneous fermentation were characterized by stronger yeasty, fermented, and, in the case of tilia mead, alcoholic aromas. The addition of *G. geotrichum* during fermentation noticeably increased the floral and rum sensation in acacia mead, floral and fermented in buckwheat mead, and rum, alcoholic, and honey in tilia meads.

### 2.2. Odor-Active Compounds Identification

To understand which specific compounds are responsible for the characteristic aroma of meads and what makes a product attractive to consumers, a screening for odor-active compounds was performed. In the first stage of the research, all twelve safe extracts were subjected to GC–O analysis. The application of GC–O analyses showed a total of 23 odor-active compounds in twelve tested mead samples. All identified odor-active compounds are listed in Table 1, and they are consistent with those obtained in our previous study [13]. 2- and 3-Methyl-1-butanal (malty), 2- and 3-methylbutan-1-ol (malty, solvent-like), ethyl octanoate (fruity), acetic acid (vinegar-like), 3-(methylsulfanyl)propanal (cooked potatoes-like), propanoic acid (sour, sweaty), butanoic acid (sweaty), 2-phenylacetaldehyde (honey-like), 2- and 3-methylbutanoic acid (malty, solvent-like), 2-phenylathan-1-ol (floral), 4-methylphenol (horse stable-like), phenylacetic acid (honey-like, beeswax-like), and 4-hydroxy-3-methoxybenzaldehyde (vanilla-like) were identified in all tested mead samples. Ethyl hexanoate (fruity, pineapple-like) was identified in only two mead samples undergoing spontaneous fermentation: ANS and BNS. 1-(3,4-Dihydro-2*H*-pyrrol-5-yl)ethan-1-one (roast-like) was found in only one sample (ANS). Among identified compounds, aldehydes 2- and 3-methyl-1-butanal (malty), 3-(methylsulfanyl)propanal (cooked potatoes-like), furan-2-carbaldehyde (sweet, cereal-like), 2-phenylacetaldehyde (honey-like), and 4-hydroxy-3-methoxybenzaldehyde (vanilla-like) were the biggest group together with the acids acetic acid (vinegar-like), propanoic acid (sour, sweaty), butanoic acid (sweaty), 2- and 3-methylbutanoic acid (malty, sweaty), and phenylacetic acid (beeswax-like). The third largest group was esters, ethyl pentanoate (fruity), ethyl hexanoate (fruity, pineapple-like), and ethyl octanoate (fruity), together with alcohols: 2- and 3-methylbutan-1-ol (malty, solvent-like), 3-(methylsulfynyl)propan-1-ol (cooked potatoes-like), and 2-phenylathan-1-ol (floral). Two compounds, 3-(methylsulfynyl)propan-1-ol (cooked potatoes-like) and (2*E*)-1-(2,6,6-trimethylcyclohexa-1,3-dien-1-yl)but-2-en-1-one (cooked apple-like), were identified only in meads prepared from acacia honey, in all fermentation types. 4-Methylquinazoline, an odorant with a characteristic mint-like odor was identified in all fermentation samples made from buckwheat and tilia honey but was absent in all samples prepared using acacia honey. This compound was also detected in our previous research on mead made from multifloral honey, where its concentration was highest in the honey sample, suggesting that honey may be its source [13]. The results of this study indicate that acacia honey, unlike buckwheat and tilia honey, does not contain this odor-active compound. Additionally, 3-hydroxy-4,5-dimethylfuran-2(5*H*)-one, which has a fenugreek-like odor, was identified exclusively in three samples made from buckwheat honey: BNS, BNSM, and BNY. Sweet furan-2-carbaldehyde was identified in all samples prepared from tilia honey and one sample made from buckwheat honey: BNS.

### 2.3. Odorants Quantitation

To determine the specific volatile constituents responsible for the overall aroma profile of a model meads a total of 11 compounds were quantified in response to calibration curves made for (^2^H_8_)-naphtalene as the internal standard and Headspace Solid Phase Micro-Extraction coupled with gas chromatography–time-of-flight–mass spectrometry (GC–ToF–MS) (details in Supplementary Materials). Their odor activity values (OAVs) were calculated as the ratio of their concentration to the odor threshold concentrations (OTCs) in water collected from the literature.

Odor-active compounds’ quantitation for twelve mead samples, their OTCs, and their OAVs are shown in Table 2, Table 3 and Table 4. The highest concentrations among all samples were determined for floral 2-phenylethan-1-ol, honey-like phenylacetic acid, and malty 2- and 3-methylbutanoic acid, and there are visible differences in their concentrations between all samples.

OAVs of quantitated compounds are different among tested samples. For meads made of acacia, buckwheat, and tilia honey, the highest OAVs were noted for 2- and 3-methyl-1-butanal, 2-phenylacetaldehyde, 2-phenylathan-1-ol, 2,5-dimethyl-3(2*H*)furanone, and phenylacetic acid, which are associated with odor descriptors described as malty, honey-like, floral, caramel-like, and beeswax-like. These results are in agreement with results obtained by sensory assessors, as ratings of descriptors honey, malty, and floral were rated relatively high in those meads. Despite the dominance of the same odor-active compounds in meads made of tilia, buckwheat, and acacia honeys, concentration levels for those compounds were different in all tested samples. The highest concentrations of floral 2-phenylethan-1-ol were noted for TNY and ANS meads (4.29 and 4.19 mg/L, respectively) and the lowest for BNY mead (2.0 mg/L). A high concentration of this floral odorant in the ANS sample resulted in the highest OAV for all compounds, which suggests it is the most important odorant in this type of mead. Floral 2-phenylathan-1-ol is a widely identified compound in meads and wines [18,19], and it is derived from phenylalanine in the fermentation process [20].

Visibly higher concentrations of malty, solvent-like 2- and 3-methyl-1-butanal were noted for buckwheat-based meads when compared to other mead types. The highest concentration was determined for BBY (0.0462 mg/L), followed by BNY (0.0413 mg/L), BNSM (0.0387 mg/L), and BNS (0.0334 mg/L). The differences in the concentrations of this compound are also reflected in the sensory evaluation of the discussed meads, as the notes of the malty descriptor for all buckwheat-based meads were visibly higher than those presented for other meads. Malty, solvent-like 2- and 3-methyl-1-butanal also showed the highest OAVs in all buckwheat-based meads, making it the most important and characteristic odor-active compound in meads prepared from buckwheat honey. It was also an important odor compound for two acacia-based meads, ANSM and ANY (OAV, 34 and 49, respectively), and two tilia-based meads, TNS and TNY (OAV, 44 and 60, respectively). In the rest of the tested samples, the concentration of this compound only slightly exceeded its odor threshold. The second important aldehyde identified in tested meads was 2-phenylacetaldehyde with a honey-like odor. This compound was detected in all tested mead samples, and its OAVs were comparable among all meads. The highest OAV was noted for TBY (20) and the lowest for ANSM and TNY equally (10). The concentration ranged from 0.0512 mg/L to 0.101 mg/L. Honey-like 2-phenylacetaldehyde formed during Strecker degradation is reported to be an important odorant in wines [21]. The last of quantitated aldehydes was furan-2-carbaldehyde, which was detected in one buckwheat-based mead (BNS) and three tilia-based meads (TNS, TNY, and TBY). Its concentration did not exceed its odor threshold in almost all cases (except the TBY sample, in which its OAV was equal to 1), which suggests that this compound did not have a meaningful influence on the final aroma of tested meads.

Acids represented by sweaty butanoic acid, malty and sweaty 2- and 3-methylbutanoic acid, and honey- and beeswax-like phenylacetic acid were determined in almost all tested samples (the only exception was phenylacetic acid in the TNY sample). Concentrations of butanoic acid in all tested samples were below its odor threshold concentration, but 2- and 3-methylbutanoic acid scored concentrations above its odor threshold in all buckwheat-based meads and in the ABY sample, with the highest value of 2.62 mg/L for the BNY sample. Those results are in agreement with sensory evaluation data, as all buckwheat-based meads had relatively high malty scores. Concentrations of 2- and 3-methylbutanoic acid for buckwheat-based meads are relatively higher than those obtained in our previous research for mead made from multi-floral honey (0.868 mg/L) [13]. The last of the quantified acids with a pleasant, beeswax-like odor was an important odorant in almost all tested samples (except TNY). The concentration of this compound was visibly higher for buckwheat-based meads when compared to acacia- and tilia-based meads. Higher concentrations resulted in high OAVs, making it the second most important odor-active compound in buckwheat-based meads. Those concentrations (ranging from 4.05 mg/L for the BNSM sample to 3.32 mg/L for the BBY sample) are visibly higher than those observed by Starowicz and Granvogl for meads prepared from heated and unheated wort (0.748 mg/L and 0.77 mg/L, respectively). Meads prepared from acacia and tilia honey scored concentrations of phenylacetic acid similar to or slightly higher than those observed by Starowicz and Garnvogl [11].

Caramel-like 2,5-dimethyl-3(2*H*)furanone was detected in almost all tested meads, except BNY and TNSM samples. The concentration of this compound was very diverse among samples and ranged from 0.318 mg/L (ANSM) to 1.54 mg/L (ABY). In those samples where 2,5-dimethyl-3(2)*H*-furanone was detected the concentration was above its odor threshold, and OAVs ranged from 13 (ANSM and TNS) to 62 (ABY). A high concentration of this caramel-like compound suggests that it was important for the final aroma of almost all meads. This compound is known to form easily in heated and fermented foods, and it was detected in many kinds of food products such as popcorn, non-alcoholic beverages, and wines [22].

The last group of odor-active compounds detected in tested mead samples were esters, which showed the biggest differentiation in the presence of particular compounds among all samples. Fruity ethyl octanoate was the only ester identified in all samples. Its concentration was above its odor threshold in all samples. Fruity, pineapple-like ethyl hexanoate was detected only in the ANS sample, and its concentration slightly exceeded its odor threshold (0.0052 mg/L, OAV 4). The last ester, identified in only one sample (BNS), was ethyl pentanoate, with a fruity odor. Its concentration was above the odor threshold concentration (0.00468 mg/L, OAV < 1). Additional esters (ethyl pentanoate and ethyl hexanoate) were identified only in samples fermented by wild yeasts present in acacia and buckwheat honey (spontaneous fermentation). Despite their low concentrations, not exceeding odor threshold concentrations, these compounds may affect the final aroma of the product [23].

Despite noticeable differences in aroma descriptors from sensory analysis of the prepared meads, no correlation to specific odor-active compounds was found when *G. geotrichum* was added. The strongest differences described by sensory assessors were shown for acacia-based meads, which may be explained by their subtle and delicate aroma, which is not overshadowed by dominant sensory descriptors. Further advanced research is necessary to elucidate the interactions in this context.

In summary, the observed differences in the aroma profiles of various meads could be attributed not to the diversity of aromatic compounds but rather to variations in their concentrations within specific groups of compounds. Consequently, the obtained results suggest that the identification of odor-active compounds for each individual product might be unnecessary. Instead, focusing on the primary product might be sufficient, with determining differences between variants through quantitative analysis alone. This approach may allow for the efficient identification of differences in aroma profiles through quantitative analysis, without the need for exhaustive compound identification for every product.

### 2.4. Effect of Honey Varieties, Fermentation Techniques, and Production Process on the Sensory Attributes and Important Odorants Formation

**Figure 4:**
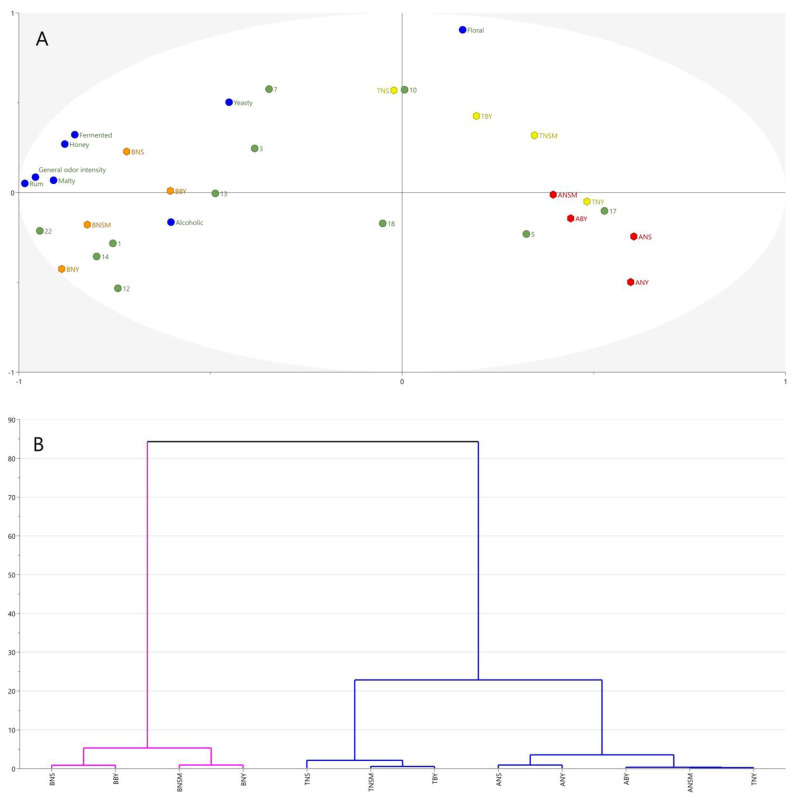
Multivariate analysis of odorant concentrations and sensory attributes of meads. The results of the multivariate analysis of important odorant concentrations and sensory attributes of the meads obtained with different technologies. (**A**) Biplot from principal component analysis (PCA). PC1 and PC2 explain 58% of the variance. Different colors represents different mead samples. (**B**) Dendrogram from hierarchical analysis. Different colors represents different clusters.

To determine the effect of technological parameters on the sensory attributes and important aroma compounds of twelve tested meads, an unsupervised principal component analysis (PCA) was applied. Multivariate analysis confirmed that the type of honey had the most significant impact on the sensory properties of mead. Those results are in agreement with Li and Sun (2019), who observed significant differences in the flavor volatiles in meads prepared with vitex, acacia, linden, multi-floral, and jujube honey [1]. The clear clustering was achieved for the analyzed data (Figure 4A). Buckwheat-based meads, irrespective of the processing type, were positioned on the left side of the plot, while tilia- and acacia-based meads were located on the right side. Interestingly, there was no clear separation between acacia- and tilia-based meads. No distinct dependencies or trends for fermentation type or heating process implementation were observed. Opposite observations were noted by Pereira et al. (2019). When two strains of *S. cerevisiae* (QA23 and ICV D47) in free and immobilized form were compared, significant differences in some characteristics of the products, such as pH, fructose concentration, volatile acidity, and volatile compound concentrations, were observed [24]. Czabaj and Rygielska (2017) and Starowicz and Granvogl (2022) also discerned that heating of mead wort results in important changes in antioxidant activity and aroma quality [11,12]. These differences may be due to the fact that, so far, all studies have focused on determining the impact of only one factor on the mead’s volatiles at a given time. This shows that, only by comparing all key variables in the production process, the real impact of these variables on the differences in the aroma of mead can be correctly identified.

**Figure 5:**
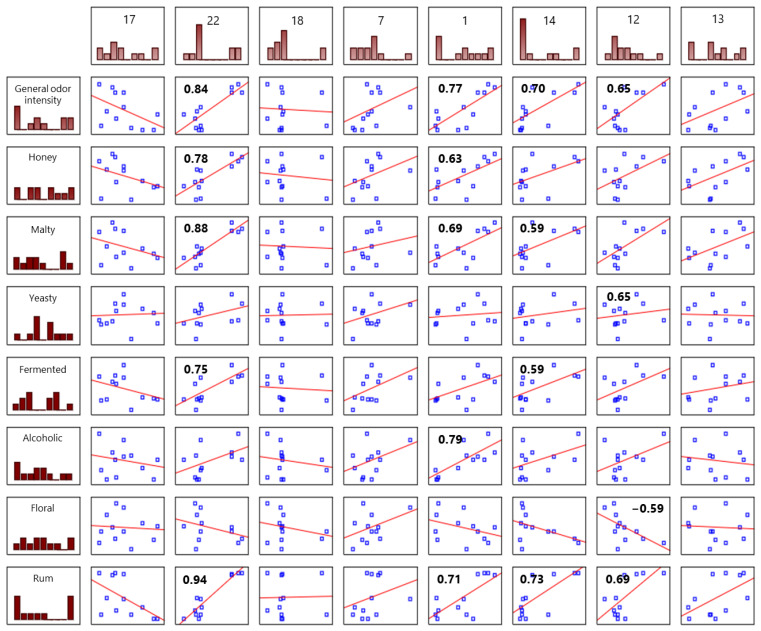
Correlations between odorant concentrations and sensory attributes. The results of correlation analysis between the important odorant concentrations and sensory attributes obtained using the Pearson correlation coefficient test. Exact values are given only for significant correlations (*p*-value < 0.05).

BNS mead was the most associated with fermented, honey, malty, rum, and general odor intensity attributes, while BNY and BNSM were associated with the concentration of phenylacetic acid, 2- and 3-methylbutanal, 2- and 3-methylbutanoic acid, and butanoic acid. TNS mead was associated with the presence of furan-2-carbaldehyde. Hierarchical analysis confirmed the clustering of the analyzed meads into two groups (Figure 4B). Interestingly, TNY was more similar to acacia-based meads than to other tilia-based products (Figure 5). These meads were associated with the concentration of 2-phenylethan-1-ol and ethyl hexanoate.

Moreover, to confirm the effect of individual important odorants on the perception of odor attributes, a correlation analysis between sensory attributes and the concentration of each important odorant was performed. General odor intensity was strongly correlated with the concentrations of phenylacetic acid, 2- and 3-methyl-1-butanal, 2- and 3-methylbutanoic acid, and butanoic acid. These compounds (except sweaty butanoic acid) showed high concentrations and high OAV values in tested samples in all honey types used. Despite lower concentrations of butanoic acid, the correlation between overall aroma intensity was significant, which may suggest that this compound interacts with other odorants even in lower concentrations. These compounds also contributed to the perception of a malty aroma, with phenylacetic acid contributing the most (r = 0.88). Three of these compounds, 2- and 3-methyl-1-butanal, 2- and 3-methylbutanoic acid, and phenylacetic acid, are connected with odors that are honey-like, beeswax-like, and malty. The perception of a honey odor was strongly associated with the concentrations of phenylacetic acid and 2- and 3-methyl-1-butanal. Both compounds are produced mainly during the fermentation and maturation processes [25,26]. The perception of fermented aroma was strongly correlated with compound phenylacetic acid (r = 0.75) and mildly correlated with compound 2- and 3-methylbutanoic acid (r = 0.59). The perception of an alcoholic odor was associated with the concentration of compound 2- and 3-methyl-1-butanal (r = 0.79). Notably, a negative correlation was observed between a floral aroma and butanoic acid.

## 3. Materials and Methods

### 3.1. Honey Samples

Three types of honey, acacia (*Robinia pseudoacacia* L.), buckwheat (*Fagopyrum Mill*.), and tilia (*Tilia*), were purchased from local beekeepers in the Wielkopolska region. The botanical origin of each honey was confirmed by pollen analysis according to the criteria described in Polish standard [27]. PN-88/A-77626 “Miód pszczeli”. Honey samples were stored at 4 °C until the mead preparation.

### 3.2. Yeast and Mold Preparation

Commercially available yeasts *S. cerevisiae* BC s103 (Fermentis, Lesaffre, France) were prepared according to the producer’s instructions. *G. geotrichum* molds used for the fermentation were previously isolated from the Wielkopolski fried cheese produced in Poznań (Poland), and thirty-nine strains were identified by amplification of the 18S rRNA coding sequence and then lyophilized by Szudera-Kończal et al. [7]. For the experiment purposes, strain 32 was chosen, according to its ability to produce 2-phenylacetaldehyde and 2-phenylethan-1-ol in the most desirable ratio, and ready lyophilizate was used. To the 3 mL vial with mold lyophilizate containing 9 × 10^−7^ colony-forming units (CFU), distilled water was added to the volume of 4 mL right before inoculation. To the four mead batches, 1 mL (each) of hydrated lyophilizate was added.

### 3.3. Mead Preparation

**Figure 6:**
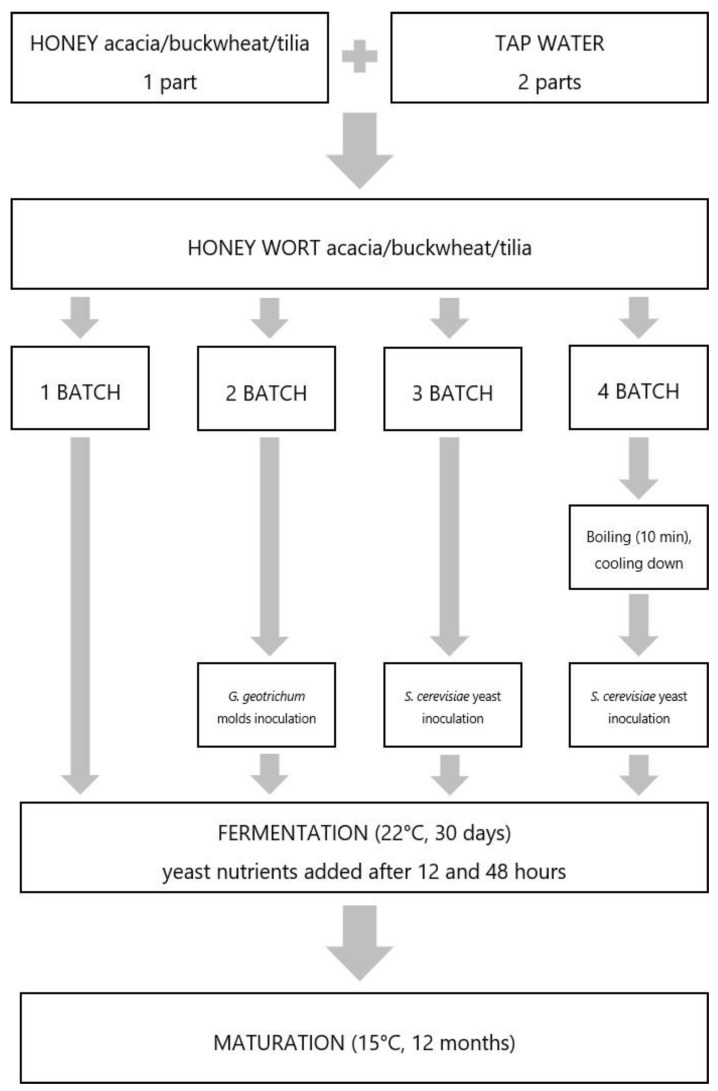
Scheme of the production process of twelve mead types.

To prepare honey wort, one part of each honey was diluted with two parts of tap water (*v*/*v*) to reach 34°Brix. From each honey, four wort batches (2 L each) were prepared: three were not heated and one batch was boiled for 10 min. After cooling the boiled batch to room temperature (around 25 °C), tap water was added to reach 34°Brix. To three unheated batches (one from every type of honey), inoculated *G. geotrichum* mold was added. To three unheated and three heated batches (two from every type of honey), *S. cerevisiae* yeast inoculum (1.5 g/L) was added. The last three batches (one from every type of honey) were not inoculated. The production process of all batches is presented in Figure 6. All wort batches were mixed and closed with a fermentation pipe. Diammonium phosphate (DAP) and the commercial nutrient “Kombi Vita” (Browin, Poland) containing diammonium hydrogen phosphate, yeast cell wall components, and thiamine hydrochloride were added to each batch in two equal parts (1 g/L and 1.5 g/L, respectively, in total) after 12 and 48 h of fermentation. Fermentation was carried out at 22 °C for 30 days. After 30 days of fermentation, meads were decanted from the yeast sediment, closed with fermentation pipes, and kept at 15 °C for 12 months for maturation. Decanting from the sediment was repeated at 3 and 6 months of maturation. Labeling of the samples was set to include honey and fermentation type and heating/not heating of the wort. All sample labels together with a precise description of the parameters used are shown in Table 5.

### 3.4. Reference Odorants

Pure reference odorants of 2- and 3-methylbutanal, butane-2,3-dione, ethyl pentanoate, 2-and 3-methylbutan-1-ol, ethyl hexanoate, ethyl octanoate, acetic acid, 3-(methylsulfanyl)propanal, furan-2-carbaldehyde, propanoic acid, butanoic acid, 2-phenylacetaldehyde, 2- and 3-methylbutanoic acid, 3-(methylsulfynyl)propan-1-ol, (2*E*)-1-(2,6,6-trimethylcyclohexa-1,3-dien-1-yl)but-2-en-1-one, 2-phenylethan-1-ol, 2,5-dimethyl-3(2*H*)furanone, 4-methylquinazoline, phenylacetic acid, and 4-hydroxy-3-methoxybenzaldehyde were purchased from Merck (Darmstadt, Germany). Compound 1-(3,4-dihydro-2*H*-pyrrol-5-yl)ethan-1-one was obtained from aromaLAB (Martinsried, Germany), and compounds 4-methylphenol and 3-hydroxy-4,5-dimethylfuran-2(5*H*)-one were obtained from Thermo Scientific (Waltham, MA, USA). (^2^*H*_8_)-naphthalene was obtained from Merck.

### 3.5. Quantitative Olfactory Profile Analysis

For all mead samples, quantitative olfactory profile analysis was performed. The sensory panel consisted of 9 experienced assessors (6 females and 3 males, aged from 25 to 55). Odor descriptors were chosen in the previous study [13]. Before the final analysis, assessors experienced a training session with the chosen odor descriptors and their descriptions. Odor descriptors were obtained from Givoudan (Vernier, Switzerland). For quantitative olfactory profile analysis, the panelists were asked to rate each odor quality using a 10 cm linear scale from 0 (not perceivable) to 10 (strongly perceivable). Samples (20 mL) were presented in covered glass vessels at room temperature. For the sensory analysis, all panelists gave their consent, and ethical permission was not required.

### 3.6. Isolation of Mead Volatiles

Meads (50 mL each) were extracted with dichloromethane (50 mL × 3, each). Combined organic phases were washed with saturated sodium chloride (150 mL × 3, each) and dried over anhydrous sodium sulfate. Nonvolatiles were removed by solvent-assisted flavor evaporation (SAFE) at 40 °C. The distillates were concentrated to a final volume of 0.5 mL using a Vigreux column (Chemland, Poland). Mead volatile isolates were stored at −20 °C until analysis. At all stages of mead volatile isolation, the odor was monitored. The extracts, distillates, and concentrated mead isolates were orthonasally tested on a strip of filter paper. The characteristic odor of mead was perceivable at all stages of the isolation of the mead’s volatiles.

### 3.7. Gas Chromatography–Olfactometry and Odor-Active Compounds Identification

A GC coupled with a cold on-column injector, a flame ionization detector (FID), a sniffing port, and DB−FFAP (30 m × 0.32 mm i.d., film thickness 0.25 μm; Agilent, Waldbronn, Germany) or DB−5 (30 m × 0.32 mm i.d., film thickness 0.25 μm; Agilent) columns was used for the identification of odor-active compounds. Identification was performed using a GC−O/FID system and a GC–ToF–MS system. Detailed specifications of the GC systems used are provided in the Supplementary Materials.

Structure proposals were developed using GC−O by the comparison of odor descriptors of detected odorants and their RIs to the data of reference compounds available in the Leibniz-LSB@TUM Odorant Database [17]. The proposed structures were confirmed by analyzing proper reference compounds in an adjusted dilution and comparing them to those present in mead volatile isolates using two columns of different polarities (DB−FFAP and DB−5). The final identification was performed by comparing the mass spectra of the compounds identified in mead volatile isolates and proper reference standards on a GC–ToF–MS system.

### 3.8. Odorant Quantitation

2- and 3-Methyl-1-butanal, ethyl pentanoate, ethyl hexanoate, ethyl octanoate, furan-2-carbaldehyde, butanoic acid, 2-phenylacetaldehyde, 2- and 3-methylbutanoic acid, 2-phenylethan-1-ol, 2,5-dimethyl-3(2*H*)-furanone, and phenylacetic acid were quantitated using HS-SPME-GC–ToF–MS, and (^2^*H*_8_)-naphthalene was used as the internal standard. A total of 5 mL of each mead sample was added to 20 mL glass vials, and 3 g of sodium chloride was added. Mead samples were spiked with the internal standard dissolved in ethanol (in concentration ranges from 0.001 to 0.1 μg/mL). Vials were closed with crimp caps, and Divinylbenzene/Carboxen/Polydimethylsiloxane (DVB/CAR/PDMS) fiber was used for the extraction. Extraction was held at 40 °C for 40 min. All analyses were performed in triplicates.

Peak areas of the target compounds and the internal standard were collected from the extracted ion chromatograms using unique quantifier ions. The concentrations of target compounds were calculated based on the area counts of the target compound peak, the area counts of the internal standard peak, the amount of the sample used, and the amount of the internal standard added. The calibration line for every target compound was obtained by the analysis of mixtures of the analyte and (^2^*H*_8_)-naphthalene in five ratios (5:1, 2:1, 1:1, 1:2, and 1:5). Details of the quantitation are available in the Supplementary Materials (Table S1).

### 3.9. Statistical Analysis

All the analyses were conducted in triplicates unless stated differently. The differences between the study groups were compared using one-way ANOVA with Fisher’s LSD test as a post hoc, after testing the normality using the Shapiro–Wilk test. The differences with a *p*-value < 0.05 were considered significant. Correlations between parameters were analyzed using the Pearson correlation coefficient test. All the statistical analyses were performed using STATISTICA version 13.3 (TIBCO Software Inc., Palo Alto, CA, USA) software.

An unsupervised principal component analysis (PCA) was applied to classify the analyzed mead samples and to evaluate the effect of technological parameters on the sensory attributes and important aroma compounds. The PCA model was built with the SIMCA 17 software package (Umietrics, Umea, Sweden).

## 4. Conclusions

The findings from the presented research provide valuable insights into the complex interactions between honey type, wort heating, and fermentation methods in shaping the aroma and sensory characteristics of mead. By systematically investigating the impact of these three critical factors—honey type (acacia, buckwheat, and linden), wort heating, and fermentation techniques (commercial Saccharomyces cerevisiae, spontaneous fermentation, and spontaneous fermentation with the inoculation of Galactomyces geotrichum molds)—on the formation of odor-active compounds, this study contributes to a deeper understanding of mead aroma development.

The results confirmed that the type of honey plays the most significant role in determining the sensory properties of mead, with distinct clusters of aroma profiles for buckwheat, acacia, and linden honeys. Buckwheat-based meads were associated with intense honey, malty, and rum-like aromas, while acacia and linden-based meads shared a lighter, more floral profile. Despite this clear influence of honey type, no consistent trends were observed for the effects of wort heating or the fermentation method on the overall aroma, indicating that while these factors can influence specific compounds, their impact is secondary to that of honey type.

Through the use of gas chromatography–olfactometry (GC–O) and headspace SPME-GC/MS quantitation, this study identified several odor-active compounds, including phenylacetic acid, 2- and 3-methylbutanal, and butanoic acid, which were strongly correlated with sensory attributes such as honey-like, malty, and fermented aromas. Compounds like phenylacetic acid, 2- and 3-methyl-1-butanal, 2- and 3-methylbutanoic acid, and 2-phenylethan-1-ol were particularly influential in driving the perception of honey, malty, beeswax-like, and floral odors. The application of the molecular sensory science concept led to the suggestion that the identification of odor-active compounds for each individual product might be unnecessary in the case of meads. This approach may allow for efficient identification of differences in aroma profiles through quantitative analysis, without the need for exhaustive compound identification for every product. Those findings may form the basis for establishing easier and faster methods for controlling the quality parameters of meads and determining the correctness of the fermentation process by monitoring odor-active compounds. In conclusion, this research highlights the critical role of honey selection in crafting a mead’s characteristic aroma and provides mead producers with strategic guidance for optimizing sensory profiles. By understanding the contributions of odor-active compounds and their correlation with sensory attributes, producers can tailor their processes to enhance desired aromas, such as honey-like or malty notes, while controlling for less desirable characteristics.

## Figures and Tables

**Table 1:** Odor-active compounds identified among volatiles isolated from meads.

			RI ^2^		Detection in Samples											
Odorant ^1^	CAS Number	Odor Quality	FFAP	DB-5	ANS	ANSM	ANY	ABY	BNS	BNSM	BNY	BBY	TNS	TNSM	TNY	TBY
2-methyl-1-butanal	96-17-3	malty	933	683	*	*	*	*	*	*	*	*	*	*	*	*
3-methyl-1-butanal	590-86-3															
butane-2,3-dione	431-03-8	butter-like	981	621	*	*	*	nd	*	*	*	*	*	*	nd	*
ethyl pentanoate	539-82-2	fruity	1140	898	nd	nd	nd	nd	*	nd	nd	nd	*	*	nd	nd
2-methylbutan-1-ol	137-32-6	malty, solvent-like	1211	739	*	*	*	*	*	*	*	*	*	*	*	*
3-methylbutan-1-ol	123-51-3															
ethyl hexanoate	123-66-0	fruity, pineapple-like	1236	1004	*	nd	nd	nd	*	nd	nd	nd	nd	nd	nd	nd
1-(3,4-dihydro-2*H*-pyrrol-5-yl)ethan-1-one	85213-22-5	roast-like	1348	936	*	nd	nd	nd	nd	nd	nd	nd	nd	nd	nd	nd
ethyl octanoate	106-32-1	fruity	1436	1194	*	*	*	*	*	*	*	*	*	*	*	*
acetic acid	64-19-7	vinegar-like	1457	633	*	*	*	*	*	*	*	*	*	*	*	*
3-(methylsulfanyl)propanal	3268-49-3	cooked potatoes-like	1464	909	*	*	*	*	*	*	*	*	*	*	*	*
furan-2-carbaldehyde	98-01-1	sweet, cereal-like	1477	831	nd	nd	nd	nd	*	nd	nd	nd	*	*	*	*
propanoic acid	79-09-4	sour, sweaty	1535	820	*	*	*	*	*	*	*	*	*	*	*	*
butanoic acid	107-92-6	sweaty	1630	804	*	*	*	*	*	*	*	*	*	*	*	*
2-phenylacetaldehyde	122-78-1	honey-like	1650	1056	*	*	*	*	*	*	*	*	*	*	*	*
2-methylbutanoic acid	116-53-0	malty, sweaty	1670	892	*	*	*	*	*	*	*	*	*	*	*	*
3-methylbutanoic acid	503-74-2															
3-(methylsulfynyl)propan-1-ol	505-10-2	cooked potatoes-like	1724	982	*	*	*	*	nd	nd	nd	nd	nd	nd	nd	nd
(2*E*)-1-(2,6,6-trimethylcyclohexa-1,3-dien-1-yl)but-2-en-1-one	23726-93-4	cooked apple-like	1822	1387	*	*	*	*	nd	nd	nd	nd	nd	nd	nd	nd
2-phenylethan-1-ol	60-12-8	floral	1917	1119	*	*	*	*	*	*	*	*	*	*	*	*
2,5-dimethyl-3(2*H*)-furanone	3658-77-3	caramel-like	2046	1078	*	*	*	*	*	*	*	*	*	nd	*	*
4-methylphenol	106-44-5	horse stable-like	2091	1076	*	*	*	*	*	*	*	*	*	*	*	*
4-methylquinazoline	700-46-9	mint-like, foxy	2115	1357	nd	nd	nd	nd	*	*	*	*	*	*	*	*
3-hydroxy-4,5-dimethylfuran-2(5*H*)-one	28664-35-9	fenugreek-like	2213	1111	nd	nd	nd	nd	*	*	*	nd	nd	nd	nd	nd
phenylacetic acid	103-82-2	honey-like, beeswax-like	2572	1267	*	*	*	*	*	*	*	*	*	*	*	*
4-hydroxy-3-methoxybenzaldehyde	121-33-5	vanilla-like	2593	1404	*	*	*	*	*	*	*	*	*	*	*	*

(^1^) Structure assignment was based on odor quality, RIs (DB-FFAP, DB-5), and mass spectrum; data were compared with the data obtained for reference compounds analyzed under the same conditions. (^2^) Retention index, calculated by linear interpolation of retention time of the odorant and adjacent n-alkanes. *—odorant detected in the sample; nd—odorant not detected in the sample.

**Table 2:** Concentration, OTC, and OAV for odorants in acacia-based meads.

		ANS		ANSM		ANY		ABY	
Odorant	OTC ^1^ (mg/L)	Concentration ^2^ (mg/L)	OAV ^3^	Concentration (mg/L)	OAV	Concentration (mg/L)	OAV	Concentration (mg/L)	OAV
**1**	0.0005	0.00173 ^c^	3	0.0172 ^b^	34	0.0246 ^a^	49	0.000615 ^c^	1
**3**	0.015	nd	-	nd	-	nd	-	nd	-
**5**	0.0012	0.00520	4	nd	-	nd	-	nd	-
**7**	0.0087	0.00309 ^b^	<1	0.00142 ^c^	<1	0.00085 ^c^	<1	0.00624 ^a^	<1
**10**	3	nd	-	nd	-	nd	-	nd	-
**12**	2.4	0.553 ^a^	<1	0.391 ^b^	<1	0.339 ^b,c^	<1	0.267 ^c^	<1
**13**	0.0052	0.0744 ^a^	14	0.0512 ^b^	10	0.0693 ^a^	13	0.0702 ^a^	13
**14**	0.49	0.0144 ^b^	<1	0.0249 ^b^	<1	0.101 ^b^	<1	1.26 ^a^	3
**17**	0.14	4.19 ^a^	30	3.71 ^a,b^	26	3.27 ^b^	23	2.05 ^c^	15
**18**	0.025	0.351 ^b^	14	0.318 ^b^	13	0.352 ^b^	14	1.54 ^a^	62
**22**	0.068	1.10 ^a,b^	16	1.28 ^a^	19	0.902 ^b^	13	1.19 ^a^	17

(^1^) Odor threshold concentration (OTC) in water. OTCs according to Leibniz-LSB@TUM Odorant Database [17]. (^2^) Concentration (mg/L), mean of triplicates, and coefficients of variation of all odorants were <20% and, together with quantitation details, are given in the Supplementary Materials (Table S2); nd—not detected; different letters in superscript in the same line indicate a significant difference (*p* < 0.05) based on the post hoc Fisher’s least significant difference (LSD) test. (^3^) Odor activity value, calculated as the ratio of the concentration in mead to OTC.

**Table 3:** Concentration, OTC, and OAV for odorants in buckwheat-based meads.

		BNS		BNSM		BNY		BBY	
Odorant	OTC ^1^ (mg/L)	Concentration ^2^ (mg/L)	OAV ^3^	Concentration (mg/L)	OAV	Concentration (mg/L)	OAV	Concentration (mg/L)	OAV
**1**	0.0005	0.0334^b^	67	0.0387 ^a,b^	77	0.0413 ^a,b^	83	0.0462 ^a^	92
**3**	0.015	0.00468	<1	nd	-	nd	-	nd	-
**5**	0.0012	nd	-	nd	-	nd	-	nd	-
**7**	0.0087	0.00517^c^	<1	0.00714 ^a^	<1	0.0041 ^d^	<1	0.00658 ^b^	<1
**10**	3	1.12	<1	nd	-	nd	-	nd	-
**12**	2.4	0.838 ^b^	<1	1.04 ^a^	<1	0.828 ^b^	<1	0.529 ^c^	<1
**13**	0.0052	0.0713 ^c^	14	0.0990 ^a^	19	0.0836 ^b^	16	0.0955 ^a^	18
**14**	0.49	1.54 ^b^	3	2.48 ^a^	5	2.62 ^a^	5	0.547 ^c^	1
**17**	0.14	2.96 ^a^	21	2.64 ^a^	19	2.00 ^b^	14	2.54 ^a^	18
**18**	0.025	0.395 ^b^	16	1.40 ^a^	56	nd	-	0.342 ^b^	14
**22**	0.068	3.33 ^b^	49	4.05 ^a^	60	3.76 ^a^	55	3.32 ^b^	49

(^1^) Odor threshold concentration (OTC) in water. OTCs according to Leibniz-LSB@TUM Odorant Database [17]. (^2^) Concentration (mg/L), mean of triplicates, and coefficients of variation of all odorants were <20% and, together with quantitation details, are given in the Supplementary Materials (Table S2); nd—not detected; different letters in superscript in the same line indicate a significant difference (*p* < 0.05) based on the post hoc Fisher’s least significant difference (LSD) test. (^3^) Odor activity value, calculated as the ratio of the concentration in mead to OTC.

**Table 4:** Concentration, OTC, and OAV for odorants in tilia-based meads.

		TNS		TNSM		TNY		TBY	
Odorant	OTC ^1^ (mg/L)	Concentration ^2^ (mg/L)	OAV ^3^	Concentration (mg/L)	OAV	Concentration (mg/L)	OAV	Concentration (mg/L)	OAV
**1**	0.0005	0.0218 ^b^	44	0.00188 ^c^	4	0.0298 ^a^	60	0.000748 ^c^	1
**3**	0.015	nd	-	nd	-	nd	-	nd	-
**5**	0.0012	nd	-	nd	-	nd	-	nd	-
**7**	0.0087	0.0138 ^a^	2	0.00326 ^c^	<1	0.00445 ^b^	<1	0.00521 ^b^	<1
**10**	3	0.617 ^b^	<1	nd	-	0.568 ^b^	<1	3.03^a^	1
**12**	2.4	0.00251 ^c^	<1	0.236 ^b^	<1	0.350 ^a^	<1	0.274 ^b^	<1
**13**	0.0052	0.0560 ^b^	11	0.0835 ^a^	16	0.0514 ^b^	10	0.101 ^a^	20
**14**	0.49	0.129 ^b^	<1	0.0760 ^c^	<1	0.265 ^a^	<1	0.251 ^a^	<1
**17**	0.14	2.99 ^b^	21	2.70 ^b^	19	4.29 ^a^	31	2.30 ^b^	16
**18**	0.025	0.334 ^b^	13	nd	-	0.390 ^a^	16	0.407 ^a^	16
**22**	0.068	1.22 ^a^	18	0.857 ^c^	13	nd	-	0.961 ^b^	14

(^1^) Odor threshold concentration (OTC) in water. OTCs according to Leibniz-LSB@TUM Odorant Database [17]. (^2^) Concentration (mg/L), mean of triplicates, and coefficients of variation of all odorants were <20% and, together with quantitation details, are given in the Supplementary Materials (Table S2); nd—not detected; different letters in superscript in the same line indicate a significant difference (*p* < 0.05) based on the post hoc Fisher’s least significant difference (LSD) test. (^3^) Odor activity value, calculated as the ratio of the concentration in mead to OTC.

**Table 5:** Mead sample labeling.

Sample Code	Honey Type	Manufacturing Process	Fermentation Type
ANS	acacia	not boiled	spontaneous
ANSM			spontaneous + *G. geotrichum* molds
ANY			*S. cerevisiae* yeasts
ABY		boiled	
BNS	buckwheat	not boiled	spontaneous
BNSM			spontaneous + *G. geotrichum* molds
BNY			*S. cerevisiae* yeasts
BBY		boiled	
TNS	tilia	not boiled	spontaneous
TNSM			spontaneous + *G. geotrichum* molds
TNY			*S. cerevisiae* yeasts
TBY		boiled	

## Data Availability

The original contributions presented in this study are included in the article/Supplementary Materials. Further inquiries can be directed to the corresponding author.

## Supplementary Materials

The following supporting information can be downloaded at: https://www.mdpi.com/article/10.3390/molecules29245913/s1, Miscellaneous Chemicals and Materials, detailed GC systems specifications, Table S1: Experimental Data of Quantitation with Internal Standard (Quantifier Ions, Calibration Lines); Table S2: Coefficients of Variance of Odorants Quantitation of Meads.
