# Supplementary material for: Influence of Honey Varieties, Fermentation Techniques, and Production Process on Sensory Properties and Odor-Active Compounds in Meads
Source: Molecules. 2024 Dec 14;29(24):5913. doi: 10.3390/molecules29245913 (PMC11677002; doi:10.3390/molecules29245913)
Supplement: Supplementary file 1 [file molecules-29-05913-s001.zip › molecules-3332942-supplementary.pdf]

Supplementary Material

Influence of Honey Varieties, Fermentation Techniques and Production Process on Sensory Properties and Odor-Active Compounds in Meads

Daria Cicha-Wojciechowicz, Natalia Drabińska, Małgorzata Anna Majcher*

Faculty of Food Science and Nutrition, Poznań University of Life Sciences, Wojska Polskiego 31, 60-624 Poznań, Poland

* Correspondence: malgorzata.majcher@up.poznan.pl; Tel.: +48618487398

Miscellaneous Chemicals and Materials. Dichloromethane, anhydrous sodium sulfate, and sodium chloride were purchased from Merck (Darmstadt, Germany).

GC–O/FID System. An HP 6890 Series gas chromatograph (Agilent, Waldbronn, Germany) was equipped with a cold on-column injector, flame ionization detector, and an ODP 2 sniffing port (Gerstel, Mülheim an der Ruhr, Germany). A DB-FFAP column (30 m × 0.32 mm i.d., film thickness 0.25 µm; Agilent, Waldbronn, Germany) or a DB-5 (30 m × 0.32 mm i.d., film thickness 0.25 µm; Agilent) was used for separation. The carrier gas was helium. The oven temperature was 40 °C for 2 min, then ramped at 6 °C/min to 230 °C (DB-FFAP) or 240 °C (DB-5), and held for 5 min. The end of the capillary column was connected to a glass Y-splitter which divided the column effluent at a 1:1 volume ratio. One part of the effluent was transferred to the FID, while the other portion was routed to the sniffing port. During a GC-O analysis, the assessor placed the nose closely above the sniffing port and evaluated the odor of the effluent. Odor-active regions were marked and described by the assessor in the chromatogram saved on the computer disc. Linear retention indices (RIs) of the odor-active compounds were calculated after co-injection with a mixture of n-alkanes from the retention times of the odorants and the adjacent two n-alkanes by linear interpolation.

GC-ToF-MS. A 7890B gas chromatograph (Agilent) was equipped with a CIS4 injector (Gerstel), and a GC capillary column SLB-5ms (30 m × 0.25 mm i.d., film thickness 0.25 µm; Agilent). The carrier gas was helium at constant flow. The end of the column was connected to the MS interface (280 °C) of a Pegasus BT time-of-flight mass spectrometer (ToF–MS) (LECO, Mönchengladbach, Germany). Mass spectra were generated in the EI mode at 70 eV, in a range of 33–333 m/z, at a rate of 50 spectra/s. The oven temperature program was 40°C (2 min), then 9°C/min to 280 °C (4 min). LECO ChromaTOF software was used for data analysis.

Table S1. Experimental Data of Quantitation With Internal Standard (Quantifier Ions, Calibration Lines)

odorant	isotopically substituted standard	quantifier ions (*m*/*z*) analyte	quantifier ions (*m*/*z*) standard	calibration line equation[1]	R^2
**1**	($^2H_8$)-naphtalene	44	136	y=68.855x+0.3013	0.9895
**3**	($^2H_8$)-naphtalene	101	136	y=11.007x+1.8812	0.9955
**5**	($^2H_8$)-naphtalene	99	136	y=11.994x+0.3098	0.9904
**7**	($^2H_8$)-naphtalene	127	136	y=29.154x+0.0472	0.9977
**10**	($^2H_8$)-naphtalene	96	136	y=26.23x+0.0029	0.9945
**12**	($^2H_8$)-naphtalene	60	136	y=3289x-0.4864	0.9919
**13**	($^2H_8$)-naphtalene	91	136	y=278x+0.3964	0.9806
**14**	($^2H_8$)-naphtalene	60	136	y=806.89x-0.1006	0.9980
**17**	($^2H_8$)-naphtalene	122	136	y=2129.1x-0.0055	0.9978
**18**	($^2H_8$)-naphtalene	43	136	y=57753x+23.18	0.9896
**22**	($^2H_8$)-naphtalene	91	136	y=17828x-2.7962	0.9918

([1])y=peak area standard/peak area analyte; x=concentration standard (µg/mL) / concentration analyte (µg/mL).

Table S2. Coefficients of Variance of Odorants Quantitation of Meads

	meads coefficients of variance (%)											
**odorant**	**ANS**	**ANSM**	**ANY**	**ABY**	**BNS**	**BNSM**	**BNY**	**BBY**	**TNS**	**TNSM**	**TNY**	**TBY**
**1**	5.5	12.6	8.7	2.5	10.1	7.2	17.9	4.0	6.8	8.1	7.3	4.1
**3**	-	-	-	-	8.3	-	-	-	-	-	-	-
**5**	10.4	-	-	-	-	-	-	-	-	-	-	-
**7**	4.6	5.7	8.0	9.9	7.4	2.8	4.9	5.4	6.5	0.9	2.0	16.3
**10**	-	-	-	-	10.6	-	-	-	7.1	-	15.3	12.8
**12**	6.2	16.4	17.2	15.7	17.8	14.2	12.0	6.9	11.0	6.9	1.1	18.4
**13**	14.6	11.4	5.8	1.5	5.8	7.3	11.0	0.4	0.9	14.8	5.1	16.4
**14**	0.4	1.3	2.5	19.1	18.7	8.5	10.9	9.9	12.4	2.9	1.2	11.7
**17**	14.0	14.1	11.8	9.3	3.0	14.3	12.3	0.2	0.2	1.2	16.4	14.4
**18**	15.0	4.1	16.6	3.1	4.1	4.3	-	10.7	13.7	-	3.6	3.5
**22**	7.7	17.1	6.0	4.5	5.5	4.5	9.7	2.1	5.5	1.0	-	11.5
